# Association between long-term hemoglobin variability and mortality in Korean adults: a nationwide population-based cohort study

**DOI:** 10.1038/s41598-019-53709-x

**Published:** 2019-11-21

**Authors:** Minkook Son, Sung Yang

**Affiliations:** 1 Department of Biomedical Science and Engineering, Gwangju Institute of Science and Technology, Gwangju, South Korea; 2 School of Mechanical Engineering, Gwangju Institute of Science and Technology, Gwangju, South Korea

**Keywords:** Biomarkers, Medical research, Risk factors

## Abstract

Hemoglobin variability is known to be associated with mortality in patients with chronic renal failure and cardiovascular disease. However, the effect of hemoglobin variability on mortality in the general population has not yet been studied. We aimed to investigate the association between hemoglobin variability and mortality using Korean cohort from National Health Insurance Service-Health Screening 2002–2015 database. This study was conducted on 182,757 adults who underwent more than 4 health screenings from 2002 to 2009. Hemoglobin variability was assessed by 3 indices of coefficient of variation (CV), standard deviation (SD), and variability independent of the mean (VIM). Cox proportional hazard regression analysis was performed for each index of quartile groups (Q1–Q4). The hazard ratio and 95% confidence interval^l for all-cause mortality comparing Q2, Q3 and Q4 with Q1 of hemoglobin variability CV in the multivariable adjusted model were 1.07 [0.96–1.20], 1.18 [1.06–1.31] and 1.43 [1.29–1.58] respectively. As the 5% CV, SD, and VIM increased, the hazard ratio for mortality increased by 1.08 [1.06–1.10] in the multivariable adjusted model. Hemoglobin variability is not only important predictor in patients with chronic renal failure and cardiovascular disease but could also be considered as a useful predictor of mortality in the general population.

## Introduction

In several studies regarding hemoglobin and mortality, anemia and polycythemia have been demonstrated to be associated with increased mortality, where hemoglobin levels and all-cause mortality are known to form a U-shape relationship^1,2^. In particular, anemia increases the risk of hospitalization and death in the elderly^3^ and is considered as a risk factor for myocardial infarction, heart failure, sepsis, and chronic renal failure^4–7^. In addition, anemia is a common complication in patients with chronic renal failure and hematopoietic agents such as erythropoietin-stimulating agents (ESAs) are frequently used for treatment. Previous report about patients with chronic renal failure describes that higher variability of hemoglobin increases mortality^8^. In fact, the variability of hemoglobin has been of great interest to dialysis patients, and many studies report that higher variability of hemoglobin is associated with a higher mortality rate in hemodialysis patients^7,9^. Hence the National Kidney Foundation’s Kidney Disease Outcomes Quality Initiative (KDOQI) guideline suggests maintaining a hemoglobin level of 11–12 g/dL for the treatment of anemia in patients with chronic renal failure^10^ and emphasizes reducing hemoglobin variability^11^.

The mechanisms of dysregulation of iron metabolism, impaired growth of erythroid progenitor cells, and reduced erythropoietin response due to inflammation of chronic diseases are known to cause anemia with increasing red blood cell distribution width (RDW) and fluctuation in hemoglobin levels^12^. RDW has been found to be strongly correlated with inflammatory biomarkers like high sensitivity C-reactive protein (hs-CRP) and erythrocyte sedimentation rate (ESR)^13^. RDW and hs-CRP are also known to be associated with all-cause mortality^14,15^.

Although there are many researches about RDW, and hemoglobin with an important clinical meaning to some patients is included as the basic item in the health screening, the effect of hemoglobin variability in the general population has not yet been studied and thus unclear. The objective of this study was to investigate the association between hemoglobin variability and mortality in the general population using nationwide population-based Korean cohort from National Health Insurance Service-Health Screening (NHIS-HealS) 2002–2015 database.

## Methods

### Study population

We analyzed using NHIS-HealS data in Korea^16^. The NHIS database is registered with 98% of Koreans and includes all insurance claims data. We analyzed the data using the health screening cohort, which was extracted from a random sampling of approximately 10% of the total 5 million people (age ≥ 40 years) who underwent general health screening in 2002 or 2003. The NHIS-HealS data includes eligibility data (age, sex, socioeconomics, and income level), clinical data (hospital visit, hospitalization, diagnosis, treatment, and death) and health screening data (physical measurements, blood tests, and questionnaires on lifestyle and behavior). Registrants in NHIC are recommended to receive a standardized health screening at least every 2 years. The detailed explanation of data can be found on the related website^17^.

This study was conducted on 514,859 adults who visited for health screening before 2009. We analyzed 360,927 adults underwent health screening in 2008 or 2009 (index year). From 2002 to 2009, participants that received 4 or more health screenings were included. We excluded participants with cancer or end-stage renal disease (ESRD) for whole study period (2002–2015), whose hemoglobin level could change drastically, to study the impact of hemoglobin variability on mortality in the general population. Cancer patients were defined as those including C in the diagnosis code using the Tenth Revision of International Classification of Diseases (ICD-10) codes by World Health Organization^18^. Patients of end-stage renal disease were defined using the combination of the diagnosis codes (N18-19, Z49, Z94.0 and Z99.2) and claims for dialysis (O7011, O7020, O7071, O7075)^19^. Subjects with one or more missing values were excluded and those with hemoglobin less than 10 g/dL or greater than 20 g/dL were excluded^1,2^. Finally, 182,757 participants were included in our study. More information about participants and study design can be found in Supplementary Figs. S1 and S2.

### Definition for hemoglobin variability

Hemoglobin variability was defined as the variability of measured hemoglobin levels over 4 independent measurements on health screenings. There are many methods to define hemoglobin variability. However, with no gold standard to measure variability, we used 3 representative indices of variability based on various studies that refer to variability^18–20^. We used 3 indices of variability: Standard deviation (SD), Coefficient of variation (CV), and variability independent of the mean (VIM). SD is the most widely used indicator and used in this study. As SD can be affected by the mean^21^, CV and VIM were also used. The CV was calculated as 100 x SD/mean and VIM was calculated as 100 x SD/mean^beta^. Beta is the regression coefficient based on the natural logarithm of SD over the natural logarithm of the mean^22^.

### Outcomes

Endpoint of this study was defined as death. By law, all deaths should be recorded in Statistics Korea. The study population was followed from the day of health screening at 2008 or 2009 (index year) to the day of death or December 31, 2015, whichever came first. In addition, cause-of-death using ICD-10 codes in NHIS-HealS database was extracted and investigated in participants with a death date. Death from cardiovascular disease was defined as those including I in cause-of-death code^1^ and Death from respiratory disease was defined as those including J in cause-of-death code. And death from other causes was defined as those excluding I or J in cause-of-death code.

### Definition of covariates

The considered covariates were age, gender, body mass index (BMI), smoking, alcohol consumption, exercise, income, experience of blood transfusion, hypertension, diabetes, dyslipidemia, Charlson comorbidity index (CCI) category and mean hemoglobin level. BMI was calculated by dividing body weight (kg) by the square meter of height (m^2^). Information on smoking status, drinking status, and exercise status was obtained by questionnaire. Smoking status was classified as non-smokers, ex-smokers, and smokers. Drinking status was classified as non-drinking or drinking. Regular exercise was defined as at least five exercises per week. Income level was divided into two groups based on the lower 10%. The experience of blood transfusion was defined using the claims for blood transfusion^23^. Hypertension, diabetes mellitus and dyslipidemia were defined using the criteria of previous studies^18–20^. The presence of hypertension was defined as the following criteria: 1) ICD-10 codes for hypertension (I10, I11) with at least one claim per year for prescription of an anti-hypertensive drug, or 2) systolic blood pressure ≥140 mmHg or diastolic blood pressure ≥90 mmHg. The presence of diabetes was defined as the following criteria: 1) ICD-10 codes for diabetes (E10 - E14) with at least one claim per year for prescription of an anti-diabetic drug, or 2) fasting glucose level ≥126 mg/dL. The presence of dyslipidemia was defined as the following criteria: 1) ICD-10 codes (E78) for dyslipidemia with at least one claim per year for prescription of lipid-lowering agents or 2) total cholesterol level ≥240 mg/dL. The CCI was calculated based on the preexisting disease including myocardial infarction, congestive heart failure, peripheral vascular disease, cerebrovascular disease, dementia, chronic pulmonary disease, connective tissue disease, peptic ulcer, mild liver disease, diabetes with and without complications, paraplegia or hemiplegia, renal disease, any or metastatic cancer, moderate or severe liver disease, and acquired immune deficiency syndrome before the start of follow-up period^24,25^. The mean hemoglobin level was calculated by independent results of hemoglobin on health screenings.

### Statistical analysis

Baseline characteristics were presented as mean with standard deviation for continuous variables and as number with percentage (%) for categorical variables. We analyzed all participants and analyzed them by dividing to gender. The subjects of this study were categorized into four groups according to hemoglobin variability CV, SD, and VIM. Statistical analysis was performed for each index of quartile groups (Q1–Q4). The incidence rate of death was calculated by dividing the number of death cases to the total follow-up duration (person-years). The graphs for survival probability according to the quartiles of hemoglobin variability were calculated using Kaplan-Meier curves and evaluated by log-rank test. The hazard ratio (HR) and the 95% confidence interval (CI) for all-cause mortality were assessed for four groups using the Cox proportional hazards model. The proportional hazard assumption was evaluated by Schoenfeld residuals test with the logarithm of cumulative hazards function based on Kaplan-Meier estimates. There was no disturbance with the assumption of proportional hazard risk over time. The multivariable-adjusted Cox proportional hazard model was set with adjusting for age, gender, BMI, smoking, alcohol consumption, exercise, income, experience of blood transfusion, hypertension, diabetes, dyslipidemia, CCI category and mean hemoglobin level. Continuous multivariable-adjusted associations between hemoglobin variability and the HR with 95% CI for all-cause mortality were presented with restricted cubic splines. We used 4 knots at 5th, 35th, 65th, and 95th percentile according to the hemoglobin variability as suggested in the previous study^26^. And the mean values of the hemoglobin variability CV were selected as reference values (All participants: 5.0%, Male participants: 4.9%, Female participants: 5.2%) for each spline plot. As hemoglobin level can be changed in many clinical situations, we further exclude the participants in the main study with gastrointestinal (GI) bleeding, hematopoietic disorders and chronic obstructive pulmonary disease (COPD) for whole study period (2002–2015) using the definition of previous study^23^. Sensitivity analysis was performed with participants who don’t have the cancer, ESRD, GI bleeding, hematopoietic disorder and COPD. Additional sensitivity analysis was performed with participants who had received 3 or more health screenings between 2002 and 2007. A time-dependent Cox regression analysis was conducted to update for the changes in hemoglobin variability (CV) and CCI category during the follow-up period (2010–2011). In order to exclude the influence of the number of hemoglobin measurements, the number of hemoglobin measurements was fixed (n = 4) and then analyzed. The stratified analyses were performed to evaluate the potential effect modification by age, gender, smoking, experience of blood transfusion, hypertension, diabetes, dyslipidemia, CCI category and mean hemoglobin level. Likelihood ratio tests were performed for interaction testing. In subgroup analysis, smoking status was divided to non-smoker and smoker. Subgroups of CCI and mean hemoglobin levels were considered as ordinal variables and analyzed under linear assumption. Furthermore, the HR with 95% CI for cause-specific mortality divided by cardiovascular disease, respiratory disease, and other causes was calculated using the Cox proportional hazards model. In the subgroup analysis, HR and 95% CI for mortality of the highest group (Q4) was compared with the lower three groups (Q1-Q3). Statistical analyses were performed using SAS version 9.4 (SAS Institute Inc., Cary, NC, USA) and all figures were produced by R 3.6.0 (https://www.r-project.org/). The p-value < 0.05 was considered to be statistically significant.

### Ethical considerations

This study was approved by the Institutional Review Board of the Gwangju Institute of Science and Technology (20190807-EX-02-02), which waived the requirement for informed consent in regard to the anonymized data analyzed retrospectively.

## Results

The participants of this study consisted of 182,757 persons. Total of 3,332 deaths (1.8%) were identified over a median follow-up of 6.7 ± 0.7 years. The characteristics of all participants divided into four groups regarding hemoglobin variability CV were shown in Table 1. As the hemoglobin variability increased, the proportion of male gender decreased and the proportion of hypertension, diabetes, dyslipidemia and experience of blood transfusion increased. As the hemoglobin variability increased, the proportion of 3 or more CCI category and lower income increased. The proportion of smokers and alcohol consumption decreased with increasing hemoglobin variability. Mean hemoglobin levels were 13.8 to 14.2 g/dL in the four groups. The mean CVs in the four groups were 2.4%, 3.9%, 5.4%, and 8.4%, respectively. Baseline characteristics of hemoglobin variability SD and VIM were described at Supplementary Table S1. Baseline characteristics male and female participants according to the quartiles of hemoglobin variability CV were shown in Supplementary Table S2.

**Table 1:** Baseline characteristics of all participants according to the quartiles of hemoglobin variability (CV).

Total (n = 182,757)	Q1 (n = 45,690)	Q2 (n = 45,687)	Q3 (n = 45,691)	Q4 (n = 45,689)
Age (years)	56.1 ± 7.9	56.1 ± 7.8	56.7 ± 8.1	57.8 ± 8.8
Sex (male)	27,851 (61.0)	27,877 (61.0)	26,866 (58.8)	23,884 (52.3)
Body mass index (kg/m^2^)	24.1 ± 2.8	24.0 ± 2.8	24.0 ± 2.8	24.0 ± 3.0
Systolic blood pressure (mmHg)	124.6 ± 15.0	124.8 ± 15.0	125.1 ± 15.1	125.5 ± 15.5
Diastolic blood pressure (mmHg)	77.6 ± 9.9	77.8 ± 9.9	77.9 ± 9.9	77.9 ± 10.1
Fasting glucose (mg/dL)	99.4 ± 22.5	99.5 ± 23.6	99.8 ± 24.7	100.6 ± 27.0
Total cholesterol (mg/dL)	201.0 ± 35.5	201.0 ± 36.1	200.5 ± 36.9	200.0 ± 37.9
Mean hemoglobin (g/dL)	14.2 ± 1.2	14.2 ± 1.2	14.0 ± 1.2	13.8 ± 1.2
**Hemoglobin variability**				
SD (g/dL)	0.3 ± 0.1	0.6 ± 0.1	0.8 ± 0.1	1.2 ± 0.3
CV (%)	2.4 ± 0.6	3.9 ± 0.4	5.4 ± 0.5	8.4 ± 2.1
VIM (%)	25.2 ± 7.0	41.1 ± 5.0	55.8 ± 6.5	85.6 ± 20.9
Cut-off value for CV	3.3	4.6	6.3	
Hypertension	25,463 (55.7)	26,542 (58.1)	27,422 (60.0)	28,562 (62.5)
Diabetes	5,300 (11.6)	5,677 (12.4)	6,347 (13.9)	7,544 (16.5)
Dyslipidemia	15,484 (33.9)	16,228 (35.5)	16,804 (36.8)	16,532 (38.6)
Experience of blood transfusion	1938 (19.2)	2160 (21.4)	2467 (24.5)	3514 (34.9)
**Charlson Comorbidity Index**				
0	28,323 (61.9)	27,478 (60.2)	26,333 (57.6)	24,652 (53.9)
1	11,860 (26.0)	12,292 (26.9)	12,692 (27.8)	12,970 (28.4)
2	3,605 (7.9)	3,892 (8.5)	4,334 (9.5)	5,074 (11.1)
3 or more	1,902 (4.2)	2,025 (4.4)	2,332 (5.1)	2,993 (6.6)
Current smoker	8,607 (18.8)	9,216 (20.2)	8,899 (19.5)	7,947 (17.4)
Alcohol consumption	21,308 (46.6)	21,395 (46.8)	20,608 (45.1)	18,338 (40.1)
Regular exercise (5 or more times/week)	10,169 (22.3)	10,716 (23.5)	10,642 (23.3)	10,648 (23.3)
Income (lower 10%)	2,695 (5.9)	3,109 (6.8)	3,600 (7.9)	4,167 (9.1)

Abbreviation: CV, coefficient of variation; Q, quartile; SD, standard deviation; VIM, variability independent of the mean.

**Figure 1:**
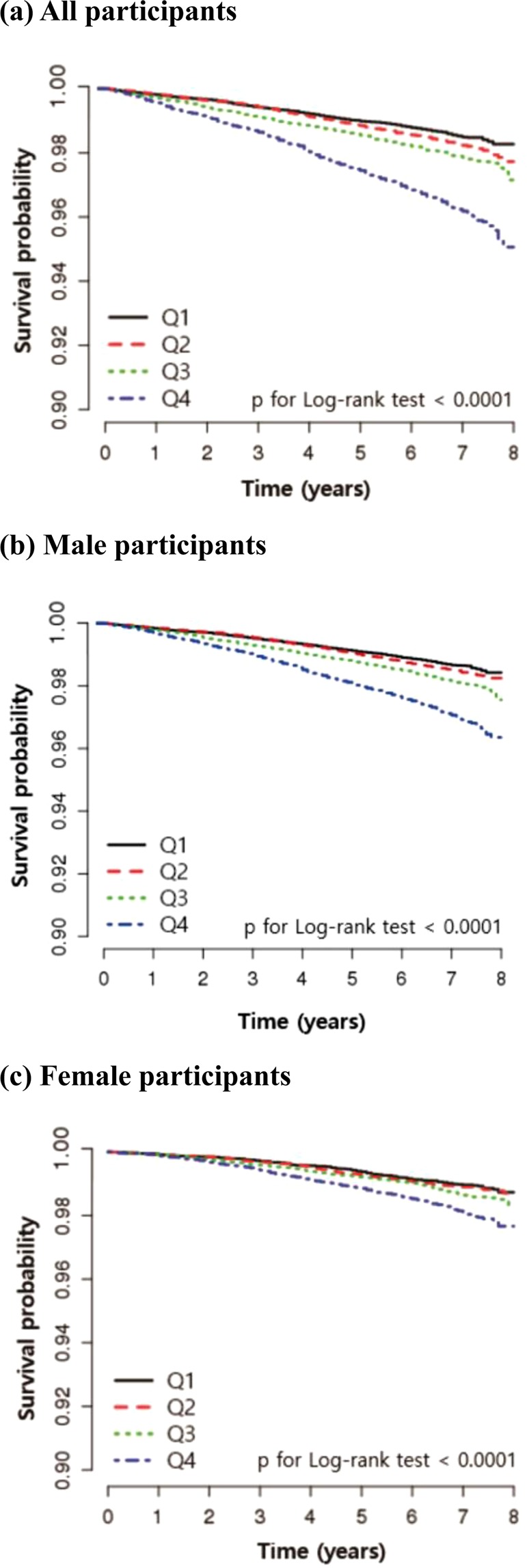
Kaplan-Meier estimates of survival probability by the quartiles of hemoglobin variability (CV). Abbreviation: CV, coefficient of variation; Q, quartile.

**Figure 2:**
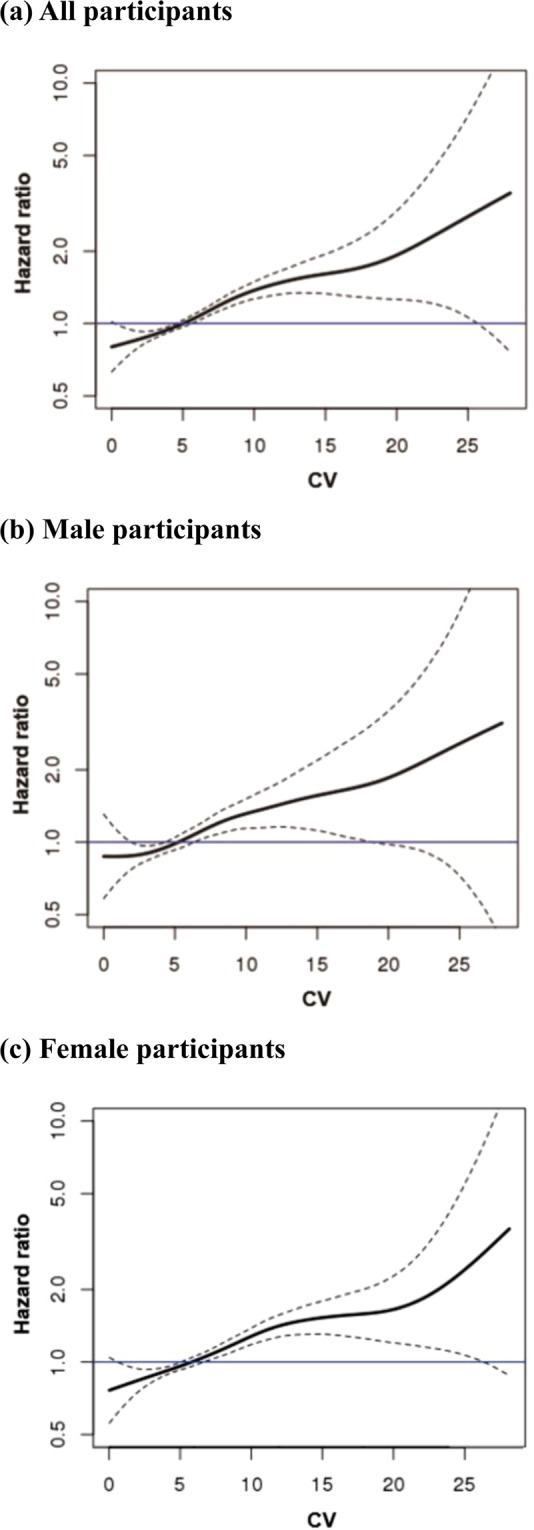
Multivariable-adjusted restricted cubic spline plots of the hazard ratio with 95% confidence interval for all-cause mortality according to hemoglobin variability (CV).

Kaplan-Meier curves and log-rank tests showed that the survival rates were progressively lower in the higher quartile at all, male and female participants (from Q1 to Q4, Fig. 1). We extracted the cause-of-death using ICD-10 code in NHIS-HealS database and identified the relatively high proportion of the highest group (Q4) in most cause-of-death (Supplementary Fig. S3). The incidence rate increased from Q1 to Q4 in all hemoglobin variability indices. Hemoglobin variability and all-cause mortality were significantly associated in the unadjusted and the multivariable adjusted Cox proportional hazard model. (Table 2) The HR and 95% CIs for mortality comparing Q2, Q3, and Q4 with Q1 of hemoglobin variability CV in the multivariable adjusted model were as follows (Q2: 1.07 [0.96–1.20], Q3: 1.18 [1.06–1.31], Q4: 1.43 [1.29–1.58]). This tendency was observed in SD and VIM as well. The HR and 95% CIs for male and female participants were shown in Supplementary Table S3. The HR and 95% CI of male participants were slightly higher than those of female participants. We also plotted the restricted cubic splines in the multivariable adjusted model to depict the linear trend of the HR with respect to hemoglobin variability. As a result, the HR for mortality increased as the value of hemoglobin variability CV increased at all, male and female participants (Fig. 2). When the hemoglobin variability indices were used as continuous variables, as the 5% CV, SD, and VIM increased, the HR and 95% CI for mortality increased by 1.08 [1.06–1.10] in the adjusted model.

**Table 2:** Hazard ratio and 95% confidence interval for all-cause mortality in all participants according to the quartiles of hemoglobin variability

	Hemoglobin variability (CV)			
	Q1	Q2	Q3	Q4
Events	581	652	805	1,294
Person-years	307,139	305,259	305,042	305,506
Incidence rate (events/1,000 person-years)	1.89	2.14	2.64	4.24
Unadjusted HR (95% CI)	1 [reference]	1.13 (1.01, 1.27)	1.40 (1.26, 1.56)	2.24 (2.03, 2.47)
Adjusted HR (95% CI)	1 [reference]	1.07 (0.96, 1.20)	1.18 (1.06, 1.31)	1.43 (1.29, 1.58)
	**Hemoglobin variability (SD)**			
	**Q1**	**Q2**	**Q3**	**Q4**
Events	586	678	786	1,282
Person-years	307,838	305,279	304,902	304,927
Incidence rate (events/1,000 person-years)	1.90	2.22	2.58	4.20
Unadjusted HR (95% CI)	1 [reference]	1.17 (1.05, 1.31)	1.36 (1.22, 1.52)	2.21 (2.01, 2.44)
Adjusted HR (95% CI)	1 [reference]	1.13 (1.01, 1.26)	1.16 (1.04, 1.29)	1.43 (1.30, 1.58)
	**Hemoglobin variability (VIM)**			
	**Q1**	**Q2**	**Q3**	**Q4**
Events	583	679	782	1,288
Person-years	307,687	305,343	304,951	304,965
Incidence rate (events/1,000 person-years)	1.89	2.22	2.56	4.22
Unadjusted HR (95% CI)	1 [reference]	1.18 (1.06, 1.32)	1.36 (1.22, 1.51)	2.23 (2.03, 2.46)
Adjusted HR (95% CI)	1 [reference]	1.13 (1.01, 1.26)	1.16 (1.04, 1.29)	1.44 (1.30, 1.59)

Abbreviation: CV, coefficient of variation; Q, quartile; HR, hazard ratio; CI, confidence interval; SD, standard deviation; VIM, variability independent of the mean.

The solid lines represent the hazard ratio and the dotted lines represent the 95% confidence interval for all-cause mortality. The horizontal blue lines correspond the normal reference hazard ratio of 1.0. The mean values of the hemoglobin variability CV were selected as reference values (All participants: 5.0%, Male participants: 4.9%, Female participants: 5.2%) for each spline plot. Abbreviation: CV, coefficient of variation.

We investigated the association between mortality and hemoglobin variability with the indices of CV, SD, and VIM and the results were all consistent. Sensitivity analysis with participants who don’t have cancer, ESRD, GI bleeding, hematopoietic disease and COPD (n = 114,207, Supplementary Table S4) was performed and the HR and 95% CIs for mortality comparing Q2, Q3, and Q4 with Q1 of hemoglobin variability CV in the adjusted model were shown as follows (Q2: 1.05 [0.87–1.26], Q3: 1.22 [1.03–1.45], Q4: 1.45 [1.23–1.71]) The further analysis was conducted for those that received more than 3 health screenings from 2002 to 2007 (n = 190,812, Supplementary Table S5), and the significant results were also observed in hemoglobin variability with mortality. The HR and 95% CIs for mortality comparing Q2, Q3, and Q4 with Q1 of hemoglobin variability CV in the adjusted model were shown as follows (Q2: 0.99 [0.91–1.08], Q3: 1.10 [1.01–1.19], Q4: 1.23 [1.14–1.33]). In time-dependent Cox regression analysis, the result was consistent (Q2: 1.03 [0.92–1.15], Q3: 1.12 [1.01–1.25], Q4: 1.39 [1.26–1.54]). In addition, the number of health screenings could act as a confounder, so we performed additional analysis on participants received only 4 times health screening (n = 102,546) and showed a significant association between hemoglobin variability and mortality. The HR and 95% CIs for mortality comparing Q2, Q3, and Q4 with Q1 of hemoglobin variability CV in the adjusted model were as follows (Q2: 1.09 [0.96–1.25], Q3: 1.21 [1.07–1.37], Q4: 1.47 [1.31–1.65]).

**Figure 3:**
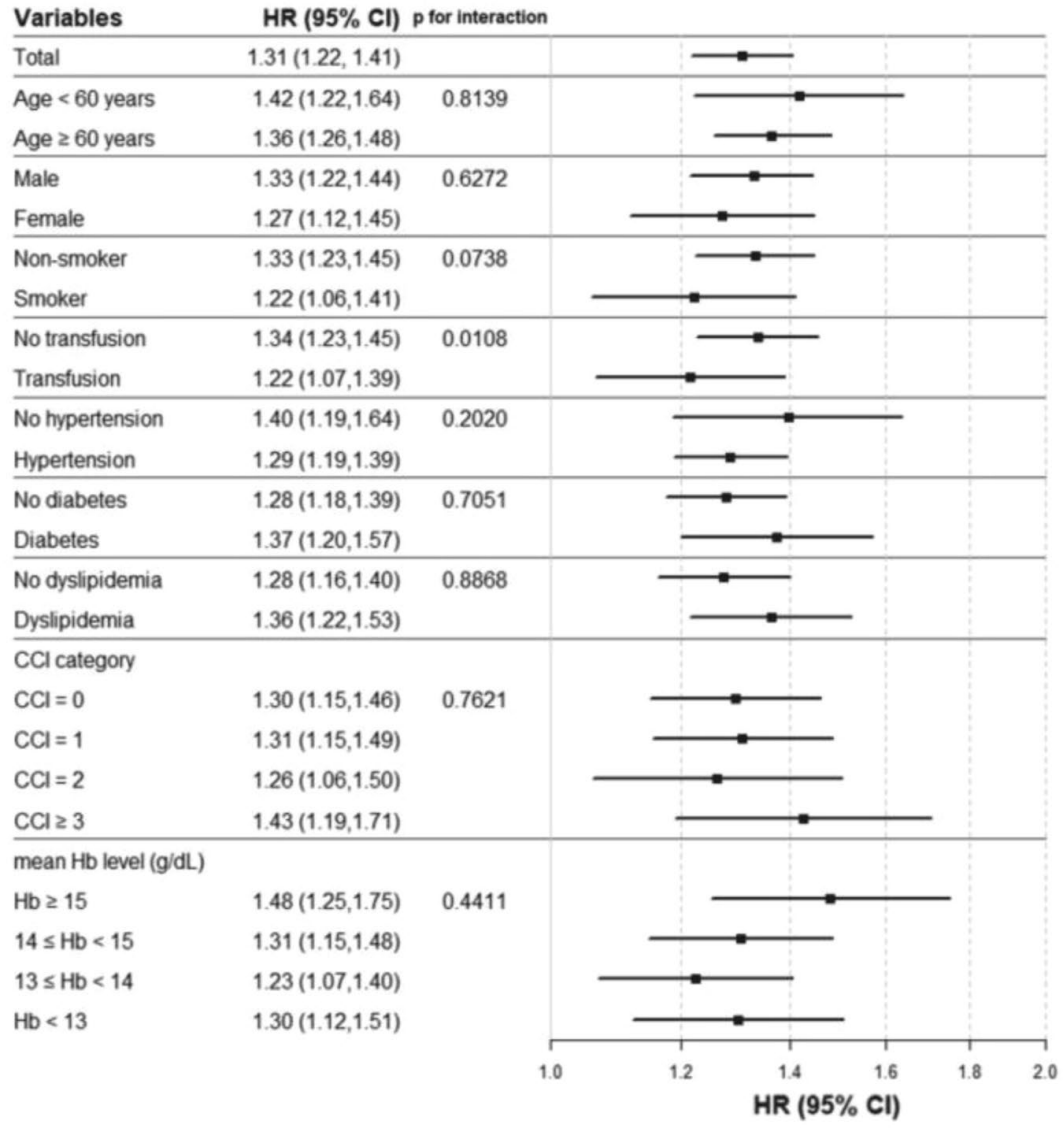
Hazard ratio and 95% confidence interval for all-cause mortality in the highest quartile (Q4) vs. lower three quartiles (Q1–Q3) of hemoglobin variability (CV) of all participants. Abbreviation: Q, quartile; CV, coefficient of variation; HR, hazard ratio; CI, confidential interval; CCI, Charlson comorbidity index; Hb, hemoglobin.

Stratification was performed according to age, gender, smoking, experience of blood transfusion, hypertension, diabetes, hyperlipidemia, CCI category, and mean hemoglobin level. In all subgroup analyses, Q4 group showed a higher HR for mortality compared to the Q1-Q3 group in the adjusted model (Fig. 3). This tendency was also consistent in other hemoglobin variability indices (Supplementary Fig. S4). And comparing Q4 group with Q1-Q3 group in the adjusted model, Q4 group had increased the HR and 95% CI of mortality from all-cause 1.31 [1.22–1.41], cardiovascular disease 1.28 [1.14–1.45], respiratory disease 1.35 [1.10–1.67], other causes 1.31 [1.19–1.44].

## Discussion

In this large-scale and long-term follow-up cohort study, we observed the association between hemoglobin variability and mortality. Previous study suggests that hemoglobin variability may affect mortality in patients with chronic renal failure, but hemoglobin may be affected by various factors^27^. Thus, this study adjusted various covariates obtained from NHIS-HealS database and examined the relationship between hemoglobin variability and mortality. Especially, CCI category was used to correct the comorbidities of participants. CCI is the most widely used comorbidity index and its validity has already been proven^24,25^. CCI was calculated based on the preexisting disease before the start of follow-up period. The stratification analysis of the CCI category revealed that higher hemoglobin variability was associated with higher mortality, regardless of whether participants had coexisting diseases or not. Furthermore, hemoglobin level and experience of transfusion also affects mortality, we performed stratified analysis by subgrouping. As a result, higher hemoglobin variability was associated with higher mortality in all subgroup of mean hemoglobin level and experience of transfusion (Fig. 3, Supplementary Fig. S4). We demonstrated that hemoglobin variability could be a useful predictor of mortality in statistical analyses. Also, through multiple sensitivity analyses, it was verified that the indices of hemoglobin variability showed that hemoglobin variability was implicated to mortality (Supplementary Tables S4, S5).

Higher hemoglobin variability is known to be associated with higher mortality in patients with chronic renal failure and cardiovascular disease. Yang W *et al*. report that hemoglobin variability in patients with chronic renal failure is associated with an increase in mortality rate of 33% for every 1 g/dL increase^28^ and Rita Ferreira *et al*. document that the prognosis in patients with acute myocardial infarction is worse with anemia or hemoglobin variability which is greater than 1.1 g/dL^29^. Furthermore, in this study, we observed that the variability of hemoglobin was also related to mortality in the general population.

The exact mechanism to explain this phenomenon has not been elucidated, but several explanations can be suggested. First, various chronic clinical conditions or diseases with inflammation can reduce the iron concentration in the body and this indicates limited iron use of erythroid precursor cells. In another study, hypoferremia and anemia progressed when Interleukin-1 (IL-1) and Tumor necrosis factor-α (TNF-α), the pro-inflammatory cytokines, were injected into rats^30^. In addition, TNF-α and Interferon-γ (IFN-γ) inhibit erythropoietin production in the kidneys, and these conditions can alter hemoglobin levels by interfering with erythropoiesis and causing anemia^12^. Inflammation can hinder erythropoiesis, consequently increasing RDW and hemoglobin variability. Kalantar-Zadeh K *et al*. suggest that inflammation is associated with protein-energy malnutrition, which may be a predictor of mortality^31^ and Li, Y. *et al*. also describe hs-CRP is correlated with all-cause mortality^15^. The second mechanism that can be explained is that myocardial cells are particularly susceptible to hemoglobin variability. Myocardial cells compensate for it by either increasing cardiac output or stimulating myocardial cell growth. These repeated stimuli can cause enlargement and hypertrophy of the ventricles^32,33^. The autonomic nervous system is also vulnerable to hemoglobin variability and this autonomic nervous system disorder may be a risk factor for sudden death, particularly in patients with chronic renal failure^34,35^. Based on these explanations, changes in hemoglobin may be a phenomenon caused by an inflammatory state and can be a factor associated with mortality by affecting myocardial cells and autonomic nervous system.

Maintaining hemoglobin at a constant level is an essential process for continued oxygen delivery to the tissue^36^. Hemoglobin in a healthy person fluctuates usually not more than 1 g/dL^37,38^. In this study, we observed hemoglobin variability SD up to 4 g/dL, and it was confirmed that the HR for mortality increased as the hemoglobin variability increased through the restricted cubic spline (Fig. 2).

Several limitations should be acknowledged in this study. First, discrepancies between performed medical practice and recorded claim data may lead to inaccurate analysis. To reduce inaccuracies, this study defined the disease with definitions used in the previous studies^18–20^. Second, hemoglobin variability can vary depending on the individual’s comorbidities, and all of these diseases can potentially become covariates. However, because we cannot assess all kinds of comorbidities, we have used CCI, the most widely used comorbidities assessment tool^24,25^. In particular, we found that hemoglobin variability is related to mortality even in the case of CCI = 0, where most of chronic diseases are excluded. Third, this study was limited to participants who completed 4 or more health screenings in the given period of time, so we cannot exclude the possibility that the selection bias worked. Forth, hemoglobin level is also related to inflammatory conditions. But, in this study, blood tests that can detect inflammation like hs-CRP and ESR were not obtained. Future studies should be supplemented by considering this limitation. Finally, since this study was conducted only for Koreans by NHIS-Heals cohort, additional studies are needed to generalize our results.

Despite these limitations, this study has several strengths. To our knowledge, this is the first large-scale study to describe the relationship between hemoglobin variability and mortality in the general population. This study analyzed the nationwide population-based Korean cohort from NHIS-HealS database with long term follow-up and revealed a significant association between hemoglobin variability and mortality. In addition, the multiple sensitivity analysis and the stratified analysis enhances the generalization of our findings.

We present the significant association between hemoglobin variability and mortality. Hemoglobin variability is not only important predictor in patients with chronic renal failure and cardiovascular disease but may also be considered as a useful predictor of mortality in the general population.

## Supplementary information

Supplementary information
