# Supplementary material for: Association between long-term hemoglobin variability and mortality in Korean adults: a nationwide population-based cohort study
Source: Sci Rep. 2019 Nov 21;9:17285. doi: 10.1038/s41598-019-53709-x (PMC6872712; doi:10.1038/s41598-019-53709-x)

**Association between long-term hemoglobin variability and mortality in Korean adults: a nationwide population-based cohort study**

**Minkook Son^1^ & Sung Yang^1,2^**

**Author Affiliations**

^1^Department of Biomedical Science and Engineering, Gwangju Institute of Science and Technology

^2^School of Mechanical Engineering, Gwangju Institute of Science and Technology

**Corresponding Author**

Sung Yang, PhD

E-mail: [syang@gist.ac.kr](mailto:syang@gist.ac.kr)

**Supplementary Table S1. Baseline characteristics of all participants according to the quartiles of hemoglobin variability (SD, VIM)**

Abbreviation: SD, standard deviation; VIM, variability independent of the mean; Q, quartile; CV, coefficient of variation.

**(a) Hemoglobin variability (SD)**

| **Total**  **(n = 182,757)** | **Q1**  **(n = 45,690)** | **Q2**  **(n = 45,688)** | **Q3**  **(n = 45,686)** | **Q4**  **(n = 45,693)** |
| --- | --- | --- | --- | --- |
| Age (years) | 56.5 ± 8.1 | 56.2 ± 7.9 | 56.5 ± 8.1 | 57.4 ± 8.7 |
| Sex (male) | 23,990 (52.5) | 26,742 (58.5) | 27,613 (60.4) | 28,133 (61.6) |
| Body mass index (kg/m^2^) | 24.0 ± 2.8 | 24.0 ± 2.8 | 24.0 ± 2.9 | 24.1 ± 2.9 |
| Systolic blood pressure (mmHg) | 124.2 ± 15.1 | 124.6 ± 15.0 | 125.2 ± 15.0 | 126.0 ± 15.4 |
| Diastolic blood pressure (mmHg) | 77.2 ± 9.9 | 77.7 ± 9.9 | 78.0 ± 9.9 | 78.4 ± 10.0 |
| Fasting glucose (mg/dL) | 98.7 ± 21.7 | 99.4 ± 23.4 | 99.9 ± 24.6 | 101.3 ± 27.9 |
| Total cholesterol (mg/dL) | 201.3 ± 35.6 | 201.1 ± 36.1 | 200.4 ± 36.9 | 199.5 ± 37.7 |
| Mean hemoglobin (g/dL) | 14.0 ± 1.2 | 14.1 ± 1.2 | 14.1 ± 1.2 | 14.1 ± 1.2 |
| **Hemoglobin variability** |  |  |  |  |
| SD (g/dL) | 0.3 ± 0.1 | 0.6 ± 0.1 | 0.8 ± 0.1 | 1.2 ± 0.3 |
| CV (%) | 2.4 ± 0.7 | 3.9 ± 0.5 | 5.4 ± 0.7 | 8.3 ± 2.1 |
| VIM (%) | 25.0 ± 6.7 | 40.9 ± 3.9 | 55.7 ± 5.0 | 86.1 ± 20.4 |
| Cut-off value for SD | 0.5 | 0.6 | 0.9 |  |
| Hypertension | 24,788 (54.3) | 26,313 (57.6) | 27,511 (60.2) | 29,377 (64.3) |
| Diabetes | 5,035 (11.0) | 5,559 (12.2) | 6,467 (14.2) | 7,807 (17.1) |
| Dyslipidemia | 15,575 (34.1) | 16,170 (35.4) | 16,645 (36.4) | 17,739 (38.9) |
| Experience of blood transfusion | 2,186 (21.7) | 2,235 (22.2) | 2,416 (24.0) | 3,242 (32.1) |
| **Charlson Comorbidity Index** |  |  |  |  |
| 0 | 27,986 (61.2) | 27,466 (60.1) | 26,397 (57.7) | 24,937 (54.6) |
| 1 | 12,021 (26.3) | 12,246 (26.8) | 12,698 (27.8) | 12,849 (28.1) |
| 2 | 3,697 (8.1) | 3,963 (8.7) | 4,281 (9.4) | 4,964 (10.9) |
| 3 or more | 1,986 (4.4) | 2,013 (4.4) | 2,310 (5.1) | 2,943 (6.4) |
| Current smoker | 7,335 (16.1) | 8,729 (19.1) | 9,129 (20.0) | 9,476 (20.7) |
| Alcohol consumption | 19,214 (42.1) | 20,826 (45.6) | 20,979 (45.9) | 20,630 (45.2) |
| Regular exercise (5 or more times/week) | 10,273 (22.5) | 10,674 (23.4) | 10,613 (23.2) | 10,615 (23.2) |
| Income (lower 10%) | 2,935 (6.4) | 3,198 (7.0) | 3,532 (7.7) | 3,906 (8.6) |

**(b) Hemoglobin variability (VIM)**

| **Total**  **(n = 182,757)** | **Q1**  **(n = 45,689)** | **Q2**  **(n = 45,689)** | **Q3**  **(n = 45,690)** | **Q4**  **(n = 45,689)** |
| --- | --- | --- | --- | --- |
| Age (years) | 56.4 ± 8.1 | 56.2 ± 7.9 | 56.5 ± 8.1 | 57.5 ± 8.7 |
| Sex (male) | 24,503 (53.6) | 26,812 (58.7) | 27,530 (60.3) | 27,633 (60.5) |
| Body mass index (kg/m^2^) | 24.0 ± 2.8 | 24.0 ± 2.8 | 24.0 ± 2.9 | 24.0 ± 2.9 |
| Systolic blood pressure (mmHg) | 124.2 ± 15.0 | 124.7 ± 15.0 | 125.2 ± 15.0 | 126.0 ± 15.4 |
| Diastolic blood pressure (mmHg) | 77.2 ± 9.9 | 77.7 ± 9.9 | 77.9 ± 9.9 | 78.3 ± 10.0 |
| Fasting glucose (mg/dL) | 98.8 ± 21.9 | 99.4 ± 23.3 | 99.9 ± 24.6 | 101.2 ± 27.8 |
| Total cholesterol (mg/dL) | 201.2 ± 35.6 | 201.1 ± 36.0 | 200.4 ± 37.0 | 199.5 ± 37.8 |
| Mean hemoglobin (g/dL) | 14.0 ± 1.2 | 14.1 ± 1.2 | 14.1 ± 1.2 | 14.0 ± 1.2 |
| **Hemoglobin variability** |  |  |  |  |
| SD (g/dL) | 0.3 ± 0.1 | 0.6 ± 0.1 | 0.8 ± 0.1 | 1.2 ± 0.3 |
| CV (%) | 2.4 ± 0.7 | 3.9 ± 0.5 | 5.4 ± 0.6 | 8.3 ± 2.1 |
| VIM (%) | 25.0 ± 6.7 | 40.9 ± 3.9 | 55.7 ± 5.0 | 86.1 ± 20.4 |
| Cut-off value for VIM | 34.1 | 47.7 | 65.1 |  |
| Hypertension | 24,864 (54.4) | 26,336 (57.6) | 27,513 (60.2) | 29,276 (64.1) |
| Diabetes | 5,068 (11.1) | 5,579 (12.2) | 6,444 (14.1) | 7,777 (17.0) |
| Dyslipidemia | 15,553 (34.0) | 16,164 (35.4) | 16,666 (36.5) | 17,746 (38.8) |
| Experience of blood transfusion | 2,158 (21.4) | 2,223 (22.1) | 2,412 (23.9) | 3,286 (32.6) |
| **Charlson Comorbidity Index** |  |  |  |  |
| 0 | 28,045 (61.4) | 27,439 (60.1) | 26,412 (57.9) | 24,890 (54.5) |
| 1 | 12,001 (26.3) | 12,240 (26.8) | 12,714 (27.8) | 12,859 (28.1) |
| 2 | 3,669 (8.0) | 3,981 (8.7) | 4,266 (9.3) | 4,989 (10.9) |
| 3 or more | 1,974 (4.3) | 2,029 (4.4) | 2,298 (5.0) | 2,951 (6.5) |
| Current smoker | 7,511 (16.4) | 8,756 (19.2) | 9,129 (20.0) | 9,273 (20.3) |
| Alcohol consumption | 19,510 (42.7) | 20,847 (45.6) | 20,930 (45.8) | 20,362 (44.6) |
| Regular exercise (5 or more times/week) | 10,244 (22.4) | 10,686 (23.4) | 10,627 (23.3) | 10,618 (23.2) |
| Income (lower 10%) | 2,896 (6.3) | 3,194 (7.0) | 3,549 (7.8) | 3,932 (8.6) |

**Supplementary Table S2. Baseline characteristics of male and female participants according to the quartiles of hemoglobin variability (CV)**

Abbreviation: CV, coefficient of variation; Q, quartile; SD, standard deviation; VIM, variability independent of the mean.

**(a) Male participants**

| **Total**  **(n = 106,478)** | **Q1**  **(n = 26,619)** | **Q2**  **(n = 26,620)** | **Q3**  **(n = 26,619)** | **Q4**  **(n = 26,620)** |
| --- | --- | --- | --- | --- |
| Age (years) | 54.8 ± 7.3 | 55.0 ± 7.3 | 55.7 ± 7.6 | 57.3 ± 8.6 |
| Body mass index (kg/m^2^) | 24.2 ± 2.7 | 24.1 ± 2.7 | 24.1 ± 2.7 | 24.0 ± 2.9 |
| Systolic blood pressure (mmHg) | 125.6 ± 14.4 | 125.9 ± 14.5 | 126.2 ± 14.5 | 126.9 ± 15.0 |
| Diastolic blood pressure (mmHg) | 78.9 ± 9.7 | 79.0 ± 9.7 | 79.1 ± 9.7 | 79.2 ± 9.9 |
| Fasting glucose (mg/dL) | 101.2 ± 23.9 | 101.7 ± 25.4 | 102.1 ± 26.8 | 103.2 ± 29.6 |
| Total cholesterol (mg/dL) | 198.2 ± 34.5 | 197.9 ± 35.2 | 197.2 ± 36.0 | 195.2 ± 36.8 |
| Mean hemoglobin (g/dL) | 15.0 ± 0.8 | 14.9 ± 0.8 | 14.8 ± 0.8 | 14.6 ± 0.9 |
| **Hemoglobin variability** |  |  |  |  |
| SD (g/dL) | 0.4 ± 0.1 | 0.6 ± 0.1 | 0.8 ± 0.1 | 1.2 ± 0.3 |
| CV (%) | 2.4 ± 0.6 | 3.8 ± 0.4 | 5.2 ± 0.5 | 8.0 ± 1.9 |
| VIM (%) | 26.3 ± 6.8 | 42.0 ± 4.3 | 56.7 ± 5.6 | 86.4 ± 20.4 |
| Cut-off value for CV | 3.2 | 4.5 | 6.1 |  |
| Hypertension | 15,852 (59.6) | 16,589 (62.3) | 17,326 (65.1) | 18,274 (68.7) |
| Diabetes | 3,564 (13.4) | 3,977 (14.9) | 4,487 (16.9) | 5,356 (20.1) |
| Dyslipidemia | 8,235 (30.9) | 8,687 (32.6) | 9,099 (34.2) | 9,502 (35.7) |
| Experience of blood transfusion | 641 (2.4) | 796 (3.0) | 881 (3.3) | 1,509 (5.7) |
| **Charlson Comorbidity Index** |  |  |  |  |
| 0 | 17,496 (65.7) | 17,025 (64.0) | 16,207 (60.9) | 14,976 (56.3) |
| 1 | 6,446 (24.2) | 6,633 (24.9) | 6,963 (26.2) | 7,249 (27.2) |
| 2 | 1,760 (6.6) | 1,957 (7.4) | 2,224 (8.4) | 2,762 (10.4) |
| 3 or more | 917 (3.5) | 1,005 (3.7) | 1,225 (4.5) | 1,633 (6.1) |
| Current smoker | 8,053 (30.3) | 8,598 (32.3) | 8,628 (32.4) | 8,544 (32.1) |
| Alcohol consumption | 17,663 (66.4) | 17,771 (66.5) | 17,443 (65.5) | 16,635 (62.5) |
| Regular exercise (5 or more times/week) | 5,783 (21.7) | 6,216 (23.4) | 6,201 (23.3) | 6,398 (24.0) |
| Income (lower 10%) | 965 (3.6) | 1,239 (4.7) | 1,533 (5.8) | 1,932 (7.3) |

**(b) Female participants**

| **Total**  **(n = 76,279)** | **Q1**  **(n = 19,067)** | **Q2**  **(n = 19,074)** | **Q3**  **(n = 19,067)** | **Q4**  **(n = 19,071)** |
| --- | --- | --- | --- | --- |
| Age (years) | 58.1 ± 8.4 | 57.8 ± 8.3 | 58.0 ± 8.6 | 58.2 ± 9.1 |
| Body mass index (kg/m^2^) | 23.9 ± 2.9 | 23.9 ± 2.9 | 23.8 ± 3.0 | 23.9 ± 3.1 |
| Systolic blood pressure (mmHg) | 122.9 ± 15.6 | 123.0 ± 15.5 | 123.4 ± 15.7 | 124.1 ± 15.9 |
| Diastolic blood pressure (mmHg) | 75.6 ± 9.8 | 75.9 ± 9.9 | 76.1 ± 9.9 | 76.5 ± 10.1 |
| Fasting glucose (mg/dL) | 96.4 ± 19.4 | 96.3 ± 20.0 | 96.6 ± 21.0 | 97.6 ± 23.5 |
| Total cholesterol (mg/dL) | 205.4 ± 36.5 | 206.0 ± 36.9 | 205.3 ± 37.9 | 204.9 ± 38.1 |
| Mean hemoglobin (g/dL) | 13.0 ± 0.7 | 13.0 ± 0.7 | 12.9 ± 0.7 | 12.8 ± 0.8 |
| **Hemoglobin variability** |  |  |  |  |
| SD (g/dL) | 0.3 ± 0.1 | 0.5 ± 0.1 | 0.7 ± 0.1 | 1.1 ± 0.3 |
| CV (%) | 2.4 ± 0.7 | 4.1 ± 0.4 | 5.6 ± 0.5 | 8.9 ± 2.1 |
| VIM (%) | 25.0 ± 6.7 | 40.9 ± 3.9 | 55.7 ± 5.0 | 86.1 ± 20.4 |
| Cut-off value for CV | 3.4 | 4.8 | 6.6 |  |
| Hypertension | 9,494 (49.8) | 9,779 (51.3) | 10,030 (52.6) | 10,645 (55.8) |
| Diabetes | 1,734 (9.1) | 1,955 (10.3) | 2,135 (11.2) | 2,563 (13.4) |
| Dyslipidemia | 7,316 (38.4) | 7,649 (40.1) | 7,652 (40.1) | 7,989 (41.9) |
| Experience of blood transfusion | 1,355 (7.1) | 1,417 (7.4) | 1,578 (8.3) | 1,902 (10.0) |
| **Charlson Comorbidity Index** |  |  |  |  |
| 0 | 10,732 (56.3) | 10,356 (54.3) | 10,180 (53.4) | 9,814 (51.5) |
| 1 | 5,483 (28.8) | 5,648 (29.6) | 5,731 (30.1) | 5,661 (29.7) |
| 2 | 1,859 (9.8) | 2,000 (10.5) | 2,082 (10.9) | 2,261 (11.9) |
| 3 or more | 993 (5.1) | 1,070 (5.6) | 1,074 (5.6) | 1,335 (6.9) |
| Current smoker | 196 (1.0) | 206 (1.1) | 193 (1.0) | 251 (1.3) |
| Alcohol consumption | 3,031 (15.9) | 3,051 (16.0) | 3,116 (16.3) | 2,999 (15.7) |
| Regular exercise (5 or more times/week) | 4,400 (23.1) | 4,469 (23.4) | 4,401 (23.1) | 4,307 (22.6) |
| Income (lower 10%) | 1,814 (9.5) | 1,918 (10.1) | 2,074 (10.9) | 2,096 (11.0) |

**Supplementary Table S3. Hazard ratio and 95% confidence interval for all-cause mortality in male and female participants according to the quartiles of hemoglobin variability**

Abbreviation: CV, coefficient of variation; Q, quartile; HR, hazard ratio; CI, confidence interval; SD, standard deviation; VIM, variability independent of the mean.

**(a) Male participants**

|  | **Hemoglobin variability (CV)** | | | |
| --- | --- | --- | --- | --- |
|  | **Q1** | **Q2** | **Q3** | **Q4** |
| Events | 375 | 445 | 542 | 980 |
| Person-years | 177,689 | 176,365 | 175,971 | 176,029 |
| Incidence rate (events/1,000 person-years) | 2.11 | 2.52 | 3.08 | 5.57 |
| Unadjusted HR (95% CI) | 1 [reference] | 1.20 (1.05, 1.38) | 1.47 (1.29, 1.67) | 2.64 (2.34, 2.97) |
| Adjusted HR (95% CI) | 1 [reference] | 1.10 (0.96, 1.26) | 1.19 (1.04, 1.35) | 1.47 (1.30, 1.66) |
|  | **Hemoglobin variability (SD)** | | | |
|  | **Q1** | **Q2** | **Q3** | **Q4** |
| Events | 401 | 464 | 514 | 936 |
| Person-years | 177,634 | 176,366 | 175,962 | 176,092 |
| Incidence rate (events/1,000 person-years) | 2.26 | 2.63 | 2.92 | 5.32 |
| Unadjusted HR (95% CI) | 1 [reference] | 1.17 (1.03, 1.34) | 1.37 (1.20, 1.56) | 2.36 (2.10, 2.65) |
| Adjusted HR (95% CI) | 1 [reference] | 1.12 (0.98, 1.28) | 1.16 (1.02, 1.32) | 1.46 (1.30, 1.65) |
|  | **Hemoglobin variability (VIM)** | | | |
|  | **Q1** | **Q2** | **Q3** | **Q4** |
| Events | 402 | 460 | 541 | 939 |
| Person-years | 177,681 | 176,304 | 175,989 | 176,080 |
| Incidence rate (events/1,000 person-years) | 2.26 | 2.61 | 3.07 | 5.33 |
| Unadjusted HR (95% CI) | 1 [reference] | 1.16 (1.02, 1.33) | 1.37 (1.20, 1.55) | 2.36 (2.10, 2.65) |
| Adjusted HR (95% CI) | 1 [reference] | 1.10 (0.96, 1.26) | 1.15 (1.01, 1.31) | 1.44 (1.28, 1.62) |

**(b) Female participants**

|  | **Hemoglobin variability (CV)** | | | |
| --- | --- | --- | --- | --- |
|  | **Q1** | **Q2** | **Q3** | **Q4** |
| Events | 194 | 206 | 241 | 349 |
| Person-years | 129,660 | 129,101 | 129,094 | 129,037 |
| Incidence rate (events/1,000 person-years) | 1.50 | 1.60 | 1.87 | 2.70 |
| Unadjusted HR (95% CI) | 1 [reference] | 1.07 (0.88, 1.30) | 1.25 (1.04, 1.51) | 1.81 (1.52, 2.16) |
| Adjusted HR (95% CI) | 1 [reference] | 1.09 (0.89, 1.32) | 1.15 (0.95, 1.39) | 1.41 (1.18, 1.69) |
|  | **Hemoglobin variability (SD)** | | | |
|  | **Q1** | **Q2** | **Q3** | **Q4** |
| Events | 197 | 216 | 240 | 337 |
| Person-years | 129,935 | 128,874 | 129,031 | 129,052 |
| Incidence rate (events/1,000 person-years) | 1.52 | 1.68 | 1.86 | 2.61 |
| Unadjusted HR (95% CI) | 1 [reference] | 1.11 (0.91, 1.35) | 1.23 (1.02, 1.48) | 1.73 (1.45, 2.06) |
| Adjusted HR (95% CI) | 1 [reference] | 1.15 (0.95, 1.40) | 1.16 (0.96, 1.40) | 1.40 (1.17, 1.67) |
|  | **Hemoglobin variability (VIM)** | | | |
|  | **Q1** | **Q2** | **Q3** | **Q4** |
| Events | 195 | 220 | 237 | 338 |
| Person-years | 129,698 | 128,997 | 129,142 | 129,056 |
| Incidence rate (events/1,000 person-years) | 1.50 | 1.71 | 1.84 | 2.62 |
| Unadjusted HR (95% CI) | 1 [reference] | 1.14 (0.94, 1.38) | 1.22 (1.01, 1.48) | 1.75 (1.46, 2.08) |
| Adjusted HR (95% CI) | 1 [reference] | 1.18 (0.97, 1.43) | 1.15 (0.95, 1.39) | 1.41 (1.18, 1.69) |

**Supplementary Table S4. Hazard ratio and 95% confidence interval for all-cause mortality (excluding the participants of cancer, ESRD, GI bleeding, hematopoietic disease and COPD, n = 114,207)**

Abbreviation: ESRD, end stage renal disease; GI bleeding, gastrointestinal bleeding; COPD, chronic obstructive pulmonary disease; CV, coefficient of variation; Q, quartile; HR, hazard ratio; CI, confidence interval; SD, standard deviation; VIM, variability independent of the mean.

|  | **Hemoglobin variability (CV)** | | | |
| --- | --- | --- | --- | --- |
|  | **Q1** | **Q2** | **Q3** | **Q4** |
| Events | 226 | 251 | 322 | 463 |
| Person-years | 191,727 | 190,371 | 190,235 | 190,905 |
| Incidence rate (events/1,000 person-years) | 1.18 | 1.32 | 1.69 | 2.43 |
| Unadjusted HR (95% CI) | 1 [reference] | 1.12 (0.94, 1.34) | 1.44 (1.21, 1.70) | 2.05 (1.75, 2.40) |
| Adjusted HR (95% CI) | 1 [reference] | 1.05 (0.87, 1.26) | 1.22 (1.03, 1.45) | 1.45 (1.23, 1.71) |
|  | **Hemoglobin variability (SD)** | | | |
|  | **Q1** | **Q2** | **Q3** | **Q4** |
| Events | 229 | 245 | 327 | 461 |
| Person-years | 191,914 | 190,537 | 190,155 | 190,632 |
| Incidence rate (events/1,000 person-years) | 1.19 | 1.29 | 1.72 | 2.42 |
| Unadjusted HR (95% CI) | 1 [reference] | 1.08 (0.90, 1.29) | 1.44 (1.22, 1.71) | 2.02 (1.72, 2.37) |
| Adjusted HR (95% CI) | 1 [reference] | 1.04 (0.87, 1.25) | 1.25 (1.05, 1.49) | 1.40 (1.19, 1.65) |
|  | **Hemoglobin variability (VIM)** | | | |
|  | **Q1** | **Q2** | **Q3** | **Q4** |
| Events | 234 | 242 | 323 | 463 |
| Person-years | 191,946 | 190,431 | 190,183 | 190,678 |
| Incidence rate (events/1,000 person-years) | 1.22 | 1.27 | 1.70 | 2.43 |
| Unadjusted HR (95% CI) | 1 [reference] | 1.04 (0.87, 1.25) | 1.39 (1.18, 1.65) | 1.99 (1.70, 2.32) |
| Adjusted HR (95% CI) | 1 [reference] | 1.00 (0.83, 1.20) | 1.20 (1.01, 1.43) | 1.38 (1.17, 1.62) |

**Supplementary Table S5. Hazard ratio and 95% confidence interval for all-cause mortality (at 2006 & 2007 index year, n = 190,812)**

Abbreviation: CV, coefficient of variation; Q, quartile; HR, hazard ratio; CI, confidence interval; SD, standard deviation; VIM, variability independent of the mean.

|  | **Hemoglobin variability (CV)** | | | |
| --- | --- | --- | --- | --- |
|  | **Q1** | **Q2** | **Q3** | **Q4** |
| Events | 1,014 | 991 | 1,219 | 1,747 |
| Person-years | 416,201 | 413,212 | 412,444 | 412,656 |
| Incidence rate (events/1,000 person-years) | 2.44 | 2.40 | 2.96 | 4.23 |
| Unadjusted HR (95% CI) | 1 [reference] | 0.99 (0.90, 1.08) | 1.22 (1.12, 1.32) | 1.74 (1.61, 1.88) |
| Adjusted HR (95% CI) | 1 [reference] | 0.99 (0.91, 1.08) | 1.10 (1.01, 1.19) | 1.23 (1.14, 1.33) |
|  | **Hemoglobin variability (SD)** | | | |
|  | **Q1** | **Q2** | **Q3** | **Q4** |
| Events | 1,022 | 1,008 | 1,215 | 1,726 |
| Person-years | 415,908 | 414,119 | 412,615 | 411,871 |
| Incidence rate (events/1,000 person-years) | 2.46 | 2.43 | 2.94 | 4.19 |
| Unadjusted HR (95% CI) | 1 [reference] | 0.99 (0.91, 1.08) | 1.20 (1.11, 1.31) | 1.71 (1.58, 1.85) |
| Adjusted HR (95% CI) | 1 [reference] | 0.97 (0.89, 1.06) | 1.08 (0.99, 1.17) | 1.22 (1.13, 1.32) |
|  | **Hemoglobin variability (VIM)** | | | |
|  | **Q1** | **Q2** | **Q3** | **Q4** |
| Events | 1,021 | 1,003 | 1,221 | 1,726 |
| Person-years | 416,537 | 413,400 | 412,335 | 412,241 |
| Incidence rate (events/1,000 person-years) | 2.45 | 2.43 | 2.96 | 4.19 |
| Unadjusted HR (95% CI) | 1 [reference] | 0.99 (0.91, 1.08) | 1.21 (1.12, 1.32) | 1.71 (1.59, 1.85) |
| Adjusted HR (95% CI) | 1 [reference] | 0.98 (0.90, 1.07) | 1.09 (1.01, 1.18) | 1.23 (1.13, 1.33) |

**Supplementary Figure S1. Flow diagram of the study**

Abbreviation: CV, coefficient of variation; SD, standard deviation; VIM, variability independent of the mean.


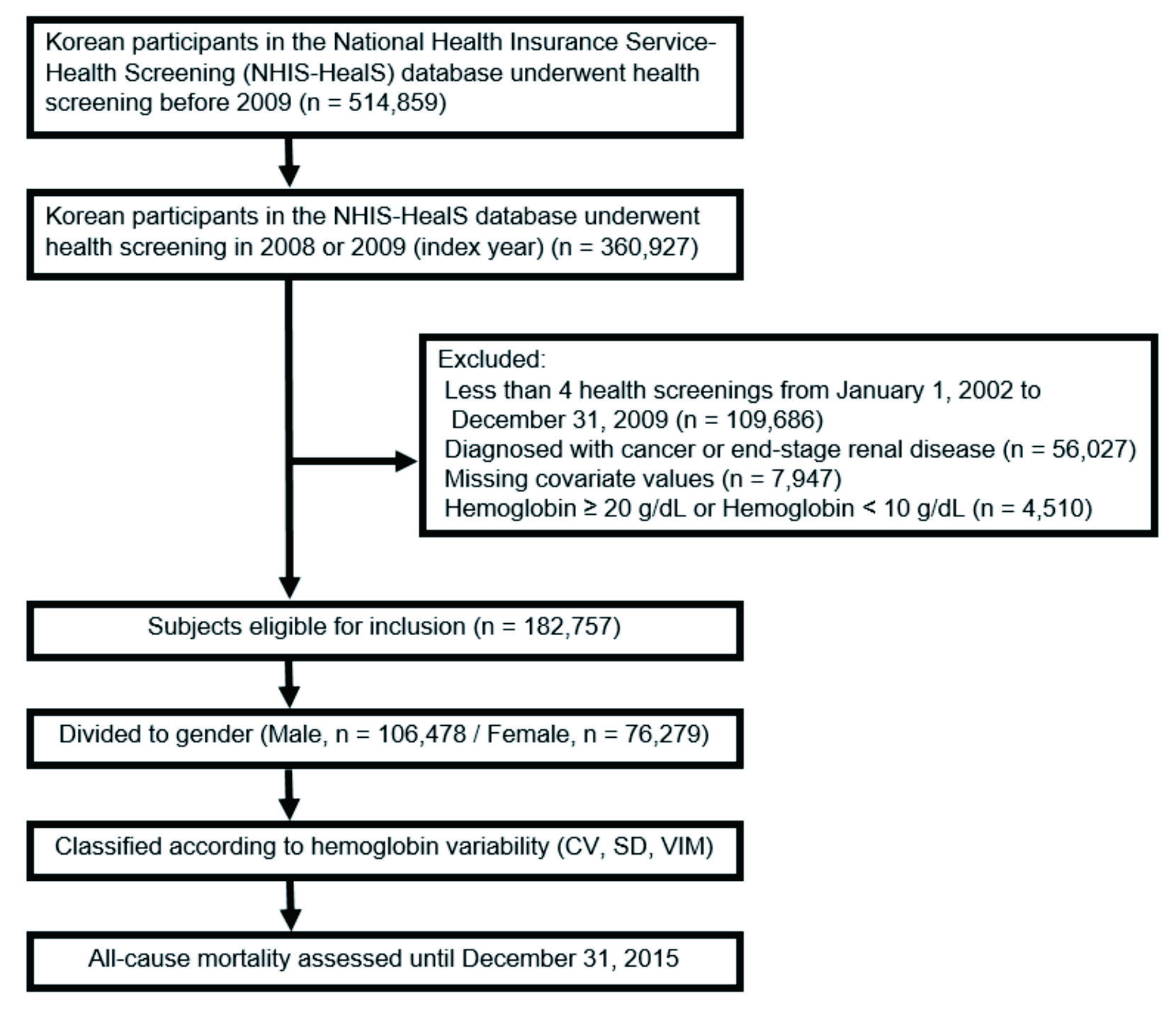


**Supplementary Figure S2. Description of the study period**


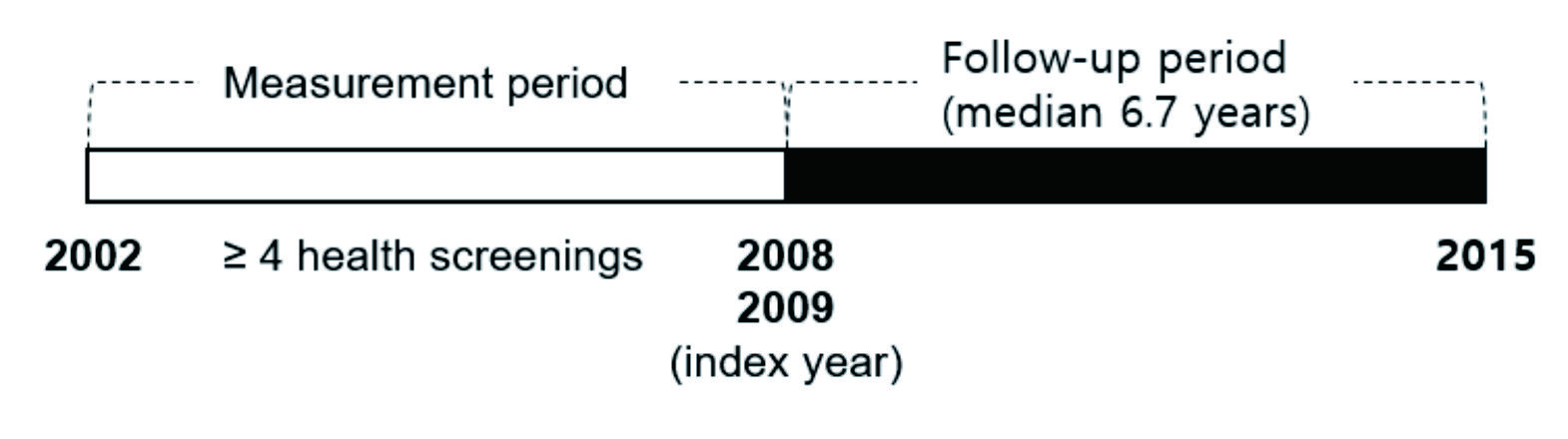


**Supplementary Figure S3. Cause-of-deaths according to the quartiles of hemoglobin variability (CV)**

Abbreviation: CV, coefficient of variation.

**(a) All participants**


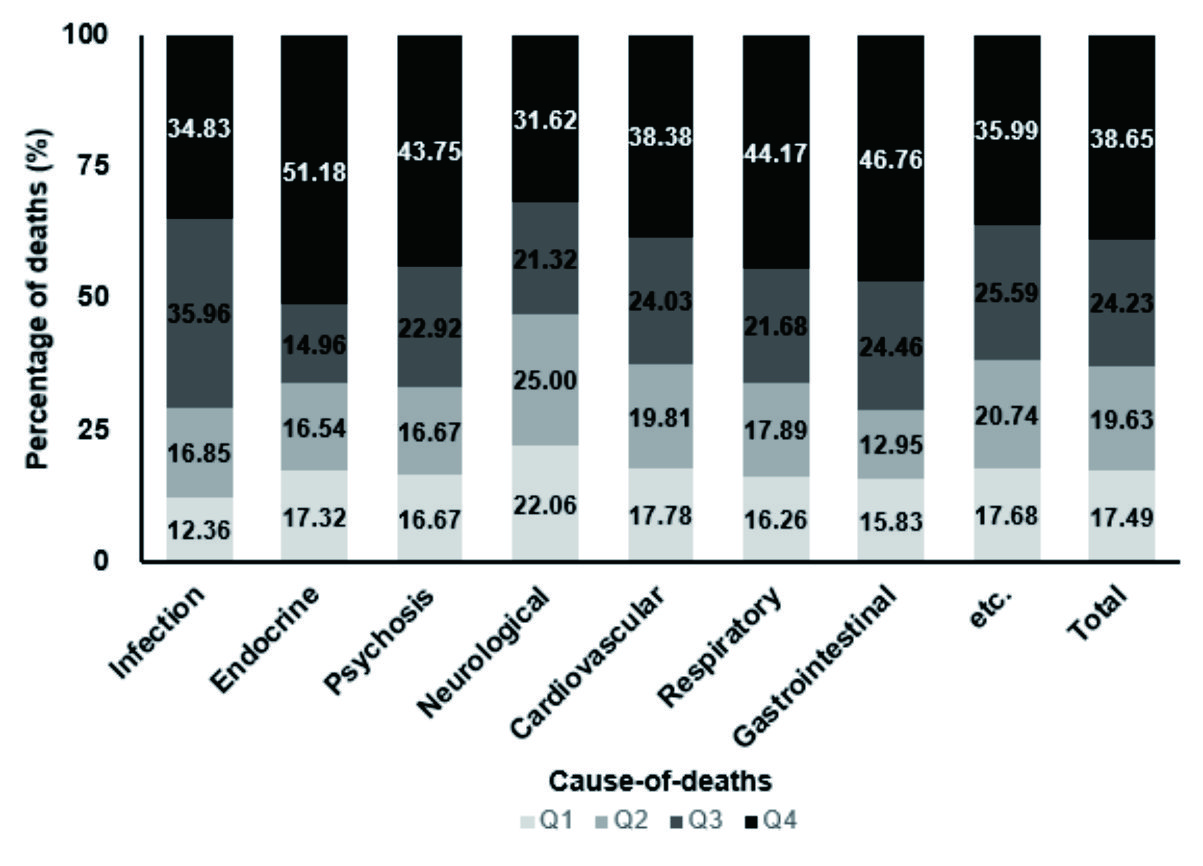


**(b) Male participants**


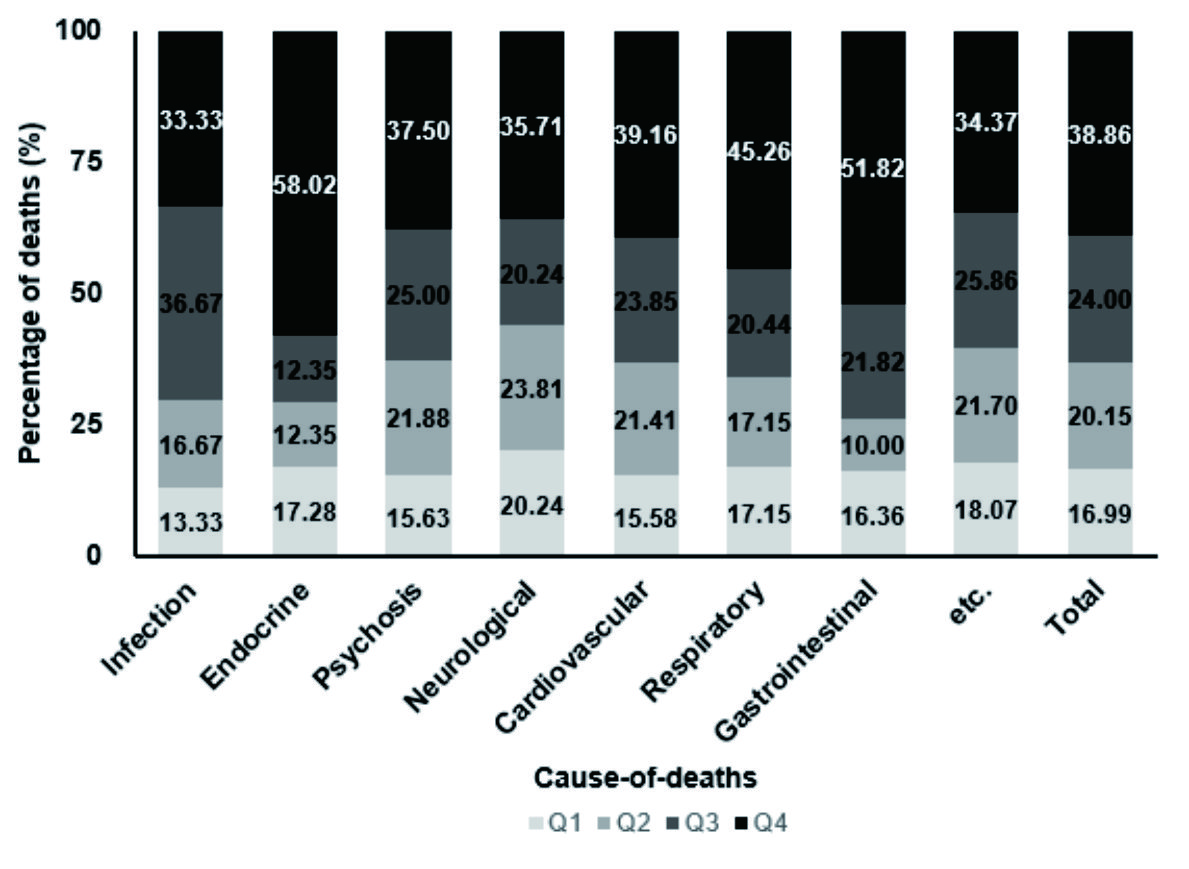


**(c) Female participants**


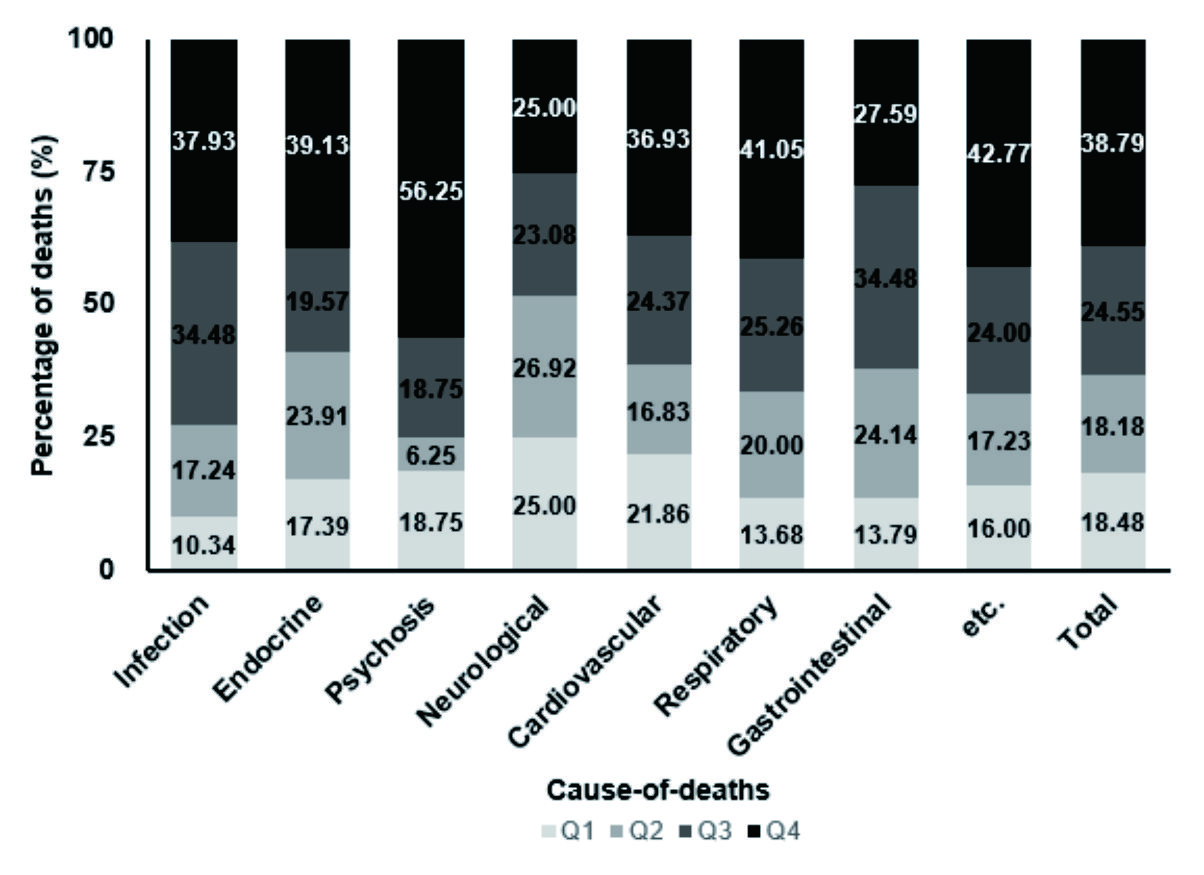


**Supplementary Figure S4. Hazard ratio and 95% confidence interval for all-cause mortality in the highest quartile (Q4) vs. lower three quartiles (Q1-Q3) of hemoglobin variability (SD, VIM) of all participants**

Abbreviation: Q, quartile; SD, standard deviation; VIM, variability independent of the mean; HR, hazard ratio; CI, confidential interval; CCI, Charlson comorbidity index; Hb, hemoglobin.

**(a) Hemoglobin variability (SD)**


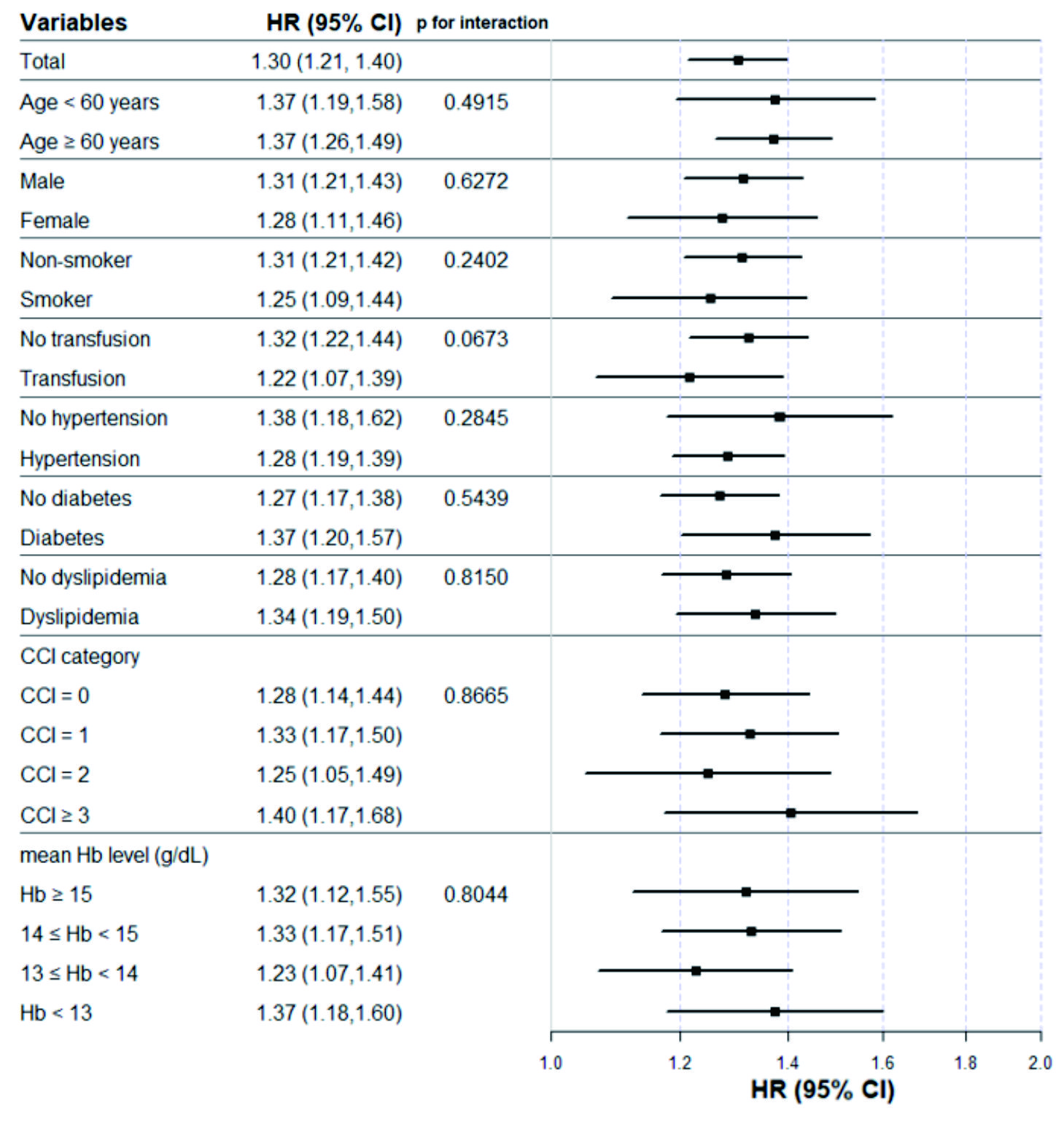


**(b) Hemoglobin variability (VIM)**


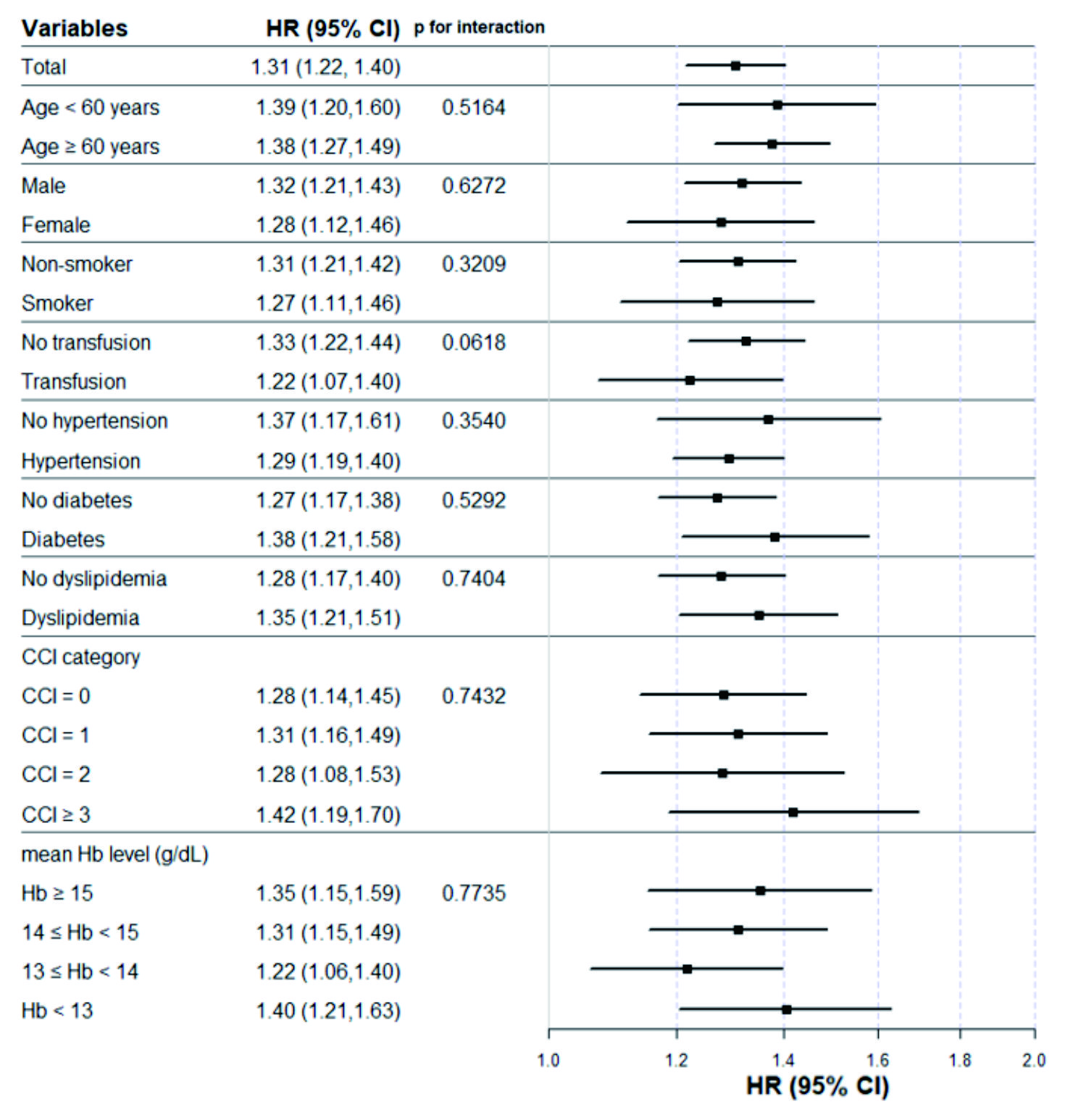

Supplement: Supplementary file 1 — Supplementary information [file 41598_2019_53709_MOESM1_ESM.docx]
